# Sturge-Weber syndrome secondary glaucoma: From Pathogenesis to Treatment

**DOI:** 10.1186/s40662-025-00432-6

**Published:** 2025-04-17

**Authors:** Tingli Wen, Lixiang Wang, Hongmei Luo, Li Tang

**Affiliations:** Department of Ophthalmology, West China Hospital of Sichuan University, Chengdu, Sichuan, 610041 People’s Republic of China

**Keywords:** Sturge-Weber syndrome, Glaucoma, Pathogenesis, Diagnosis, Drug, Surgery

## Abstract

Sturge-Weber syndrome (SWS) is a rare form of neurocutaneous disorder characterized by the involvement of neurologic, cutaneous and ocular problems. Among all SWS-related ocular abnormalities, glaucoma is the most common complication with a bimodal onset time. The occurrence of gene mutations in GNAQ has been identified as a cause of SWS. Recent studies have indicated that macrophages and mutations in GNA11 or GNB2 are also involved in the pathogenesis of SWS. Different mechanisms such as elevated episcleral venous pressure and focal venous hypertension can result in SWS secondary glaucoma (SG). In addition to glaucoma-related manifestations, SG may be associated with the typical site of facial port-wine birthmarks, choroidal vascular malformation and other ocular features. Medication and surgery are still the mainstay for SG. ROCK inhibitors have shown good performance in the control of intraocular pressure in SG but have not been verified in large sample populations. Due to the anatomical abnormalities, the incidence of surgical complications is higher. Non-penetrating surgical procedures, known for their safety and hypotensive characteristics, may be preferable instead. In general, the treatment of SG is a challenging undertaking. Early detection and treatment are crucial to preserve the visual function of patients with SWS. This review provides an overview of its pathogenesis, clinical manifestations, therapeutic agents, surgeries, and recent advances in the field of SG. The aim is to offer the latest perspectives and insights for the understanding and diagnosis of this disease.

## Background

Sturge-Weber syndrome (SWS) is a rare form of neurocutaneous disorder characterized by the involvement of neurologic, cutaneous and ocular problems. Most cases are sporadic, with an incidence between 1 in 20,000 and 1 in 50,000 [1]. So far, no geographic or ethnic correlation has been found. Manifestations of SWS are variable. The classic triad including facial capillary malformation known as port-wine birthmark (PWB), intracranial vascular malformation known as leptomeningeal hemangioma and ocular vascular malformations including conjunctival, episcleral and choroidal vascular malformations, etc.

Of all SWS-related ocular complications, the most common is glaucoma, which affects 30% to 70% of SWS patients [2]. Glaucoma is a chronic, progressive blinding disease, often associated with elevated intraocular pressure (IOP), visual field changes, and optic neuropathy. SWS secondary glaucoma (SG) is a type of refractory glaucoma with a relatively low cure rate and a higher incidence of complications [3]. Therefore, early detection and treatment are crucial for preserving the visual function of patients with SWS. However, due to the rarity of this disease, clinicians often have difficulties in understanding and diagnosing it. This review provides an overview of its pathogenesis, clinical manifestations, therapeutic agents, surgeries, and recent advances in the field of SG. The aim is to offer the latest perspectives and insights for the understanding and diagnosis of this disease.

## Pathogenesis of SWS

### Gene

Current research suggests that SWS is a disease caused by mutations in somatic genes and its clinical phenotypes are dependent on the gene involved.

Classic SWS is usually caused by mutations in the GNAQ gene, which encodes a subunit of protein G. Altered copies of GNAQ can result in either SWS or simple sporadic PWB. The severity and type are determined by when the mutation occurs, and only early mutations of GNAQ gene in the progenitor cells may lead to the development of SWS [4]. The missense mutation R183Q is the most common type of GNAQ mutation, but there are also rare variants such as the Q209R mutation [5] which can lead to different activation levels of the GNAQ gene, but all have similar downstream effects. These lead to constitutive over-activation of the Ras-Raf-MEK-ERK-mTOR pathway, resulting in aberrant cell proliferation [4]. Recent studies have also identified the presence of macrophages in brain slices from SWS patients. The macrophages adhere to endothelial cells expressing the GNAQ R183Q mutation which can produce high levels of angiogenic factor that may participate in promoting alternatively proangiogenic macrophage accumulation. Besides, macrophages may be involved in blood vessel formation and remodeling by potentially scavenging and engulfing cells and cellular debris, which may contribute to the formation of vascular malformations and the progression of SWS [6, 7].

Recent studies have broadened the phenotypic spectrum of SWS [8–10]. To distinguish it from SWS caused by mutations in the GNAQ gene (GNAQ-SWS), SWS caused by GNA11 mutations is classified as GNA11-SWS type. In Dompmartin et al.'s study, two out of five cases of GNA11-SWS were associated with glaucoma. Compared to the uniform dark red PWB of GNAQ-SWS, the PWB of GNA11-SWS were mostly patchy or reticular, with an initial lighter color that darkened over time, and with a relatively less severe neurological involvement [9].

In addition, Fjær et al. identified somatic mutations in GNB2, which encodes the β-chain of the same G-protein complex, that may be associated with SWS, as reported by deep DNA sequencing of skin biopsies, suggesting that Yes-associated protein (YAP, a transcriptional co-activator of the Hippo signaling pathway) may also be involved in the pathogenesis of SWS [11].

### Embryology

The embryological basis of SWS suggests that pathogenesis is related to neural crest damage. SWS develops as the neural crest is damaged during the third stage of the development of the primitive vascular plexus, between the fifth and eighth week of gestation, when the three structures that form the skin, eye, and head are in close proximity [12–14]. The absence of cortical bridging veins impairs venous outflow from the cerebral cortex to the dural sinuses and leads to venous stasis, elevated venous pressure, and meningeal thickening. Facial PWB may simply manifest as a cutaneous drainage pathway in response to elevated dural venous pressure [15].

### Theories of pathophysiologic mechanisms of glaucoma secondary to SWS

Glaucoma occurs in approximately 30% to 70% of patients with SWS. Several pathophysiologic mechanisms have been identified as being responsible for the development of glaucoma secondary to SWS. Anterior chamber angle deformity: in patients with SWS, the anterior chamber angle develops abnormally, resulting in increased resistance to aqueous outflow [14, 16].

1. Elevated episcleral venous pressure (EVP): EVP can manifest as a dense dilated vascular malformation outside the sclera of the affected eyes in SG. Weiss first observed that blood from SG eyes readily refluxes into Schlemm's canal and suggested that this may be due to the arterio-venous shunt leading to elevated pressure in the episcleral veins [17]. The results of EVP and ultrasound biomicroscopy in SG patients [18, 19] also support this hypothesis.
2. Focal venous hypertension: Abnormal development of the cranial veins in SWS leads to cavernous sinus hypertension, which prevents the normal outflow of ocular blood. Restriction of choroidal outflow leads to diffuse thickening, while the thickened choroid in turn rotates the iris root anteriorly, resulting in the blockage of the aqueous outflow channels. In addition, high venous pressure also increases the exudation of plasma protein exudates from the ciliary body to the iris root, and thus block the aqueous outflow channels. Parsa found increased aqueous fluorescein secretion in patients with SWS [15]. Histopathological and ultrastructural examinations of the trabecular meshwork in patients with SWS have also shown intracellular flocculent material with particulate and fine fibrous increased extracellular deposits [16, 20].
3. Other mechanisms such as premature aging of the trabecular meshwork-Schlemm's canal complex [20], and fluid hypersecretion in ciliary or choroidal capillary malformation (CM) [21].

## Diagnosis of SWS

### Staging

The onset of SG has a bimodal distribution and can be divided into early- and late-onset subtypes. Early-onset cases tend to develop between the ages of 0 to 2 years, accounting for approximately 60% of cases. The remaining 40% cases that develop mostly in late childhood or adulthood are termed late-onset [2].

Early-onset glaucoma in SWS is similar to primary congenital glaucoma (PCG) and is mainly associated with anterior chamber angle malformations. Histopathology shows a highly inserted iris, hypoplastic scleral spur, and direct attachment of the ciliary muscle to the trabecular meshwork in SWS early-onset glaucoma [14]. Mechanical stretching and thinning of the cornea often occur in congenital glaucoma, but to a lesser extent in SWS. In some studies, results showed that affected eyes of SG tend to have thicker corneas without loss of its transparency, relatively less corneal edema and Haab's striae, and lower IOP than those with PCG [22, 23]. However, in Senthil et al.’s study, corneal edema was present in 75% of the eyes at the time of diagnosing SG [24]. The discrepancy in results may be attributed to the varying age demographics of the study populations. Cornea edema is caused by the breakage of the Descemet membrane due to continuously increased IOP and globe enlargement in pediatric glaucoma [25]. Therefore, patients with earlier onset are more likely to have corneal edema and Haab's striae [26]. In addition, diffuse distribution of episcleral vessels may indicate the presence of other complicated factors than a simple anterior chamber malformation. Mixed mechanisms may coexist in the development of early-onset glaucoma. Studies suggest that the prognosis after trabeculotomy in these patients is significantly worse than those with simple anterior chamber malformation [27].

Late-onset glaucoma in SWS is mainly associated with elevated EVP. As PWB grows progressively due to vessel hypertrophy and dilation with age, there is a possibility that this pattern of vascular hypertrophy and dilatation are also occurring at the level of the episcleral veins [28, 29]. Furthermore, based on the observation of normal anterior chamber angle structure and blood within Schlemm’s canal, some studies agree that arteriovenous shunts into the episcleral vessels is a potential cause of elevated EVP [30].

SWS is commonly characterized according to Roach's classification as summarized in Table 1 [31]. There are also clinical cases of isolated choroidal CM with ipsilateral conjunctival and episcleral vascular malformations, but without glaucoma, which are considered to be one of the specific subtypes of SWS [32].

**Table 1:** Roach’s classification of Sturge-Weber syndrome

Roach’s classification	Facial involvement	Cerebral involvement	Ocular involvement
Type I	Present	Present	Feature can be observed but is not ubiquitous
Type II	Present	Absent	
Type III	Absent	Present	

### Clinical manifestations

#### Ocular presentations

SG tends to be presented as open-angle glaucoma, although cases of angle closure glaucoma caused by SWS have also been reported [33, 34]. SG with angle closure episodes has been associated with lens ectasia [35] and posterior scleritis leading to anterior rotation of the ciliary body and lens distension [36].

**Fig. 1:**
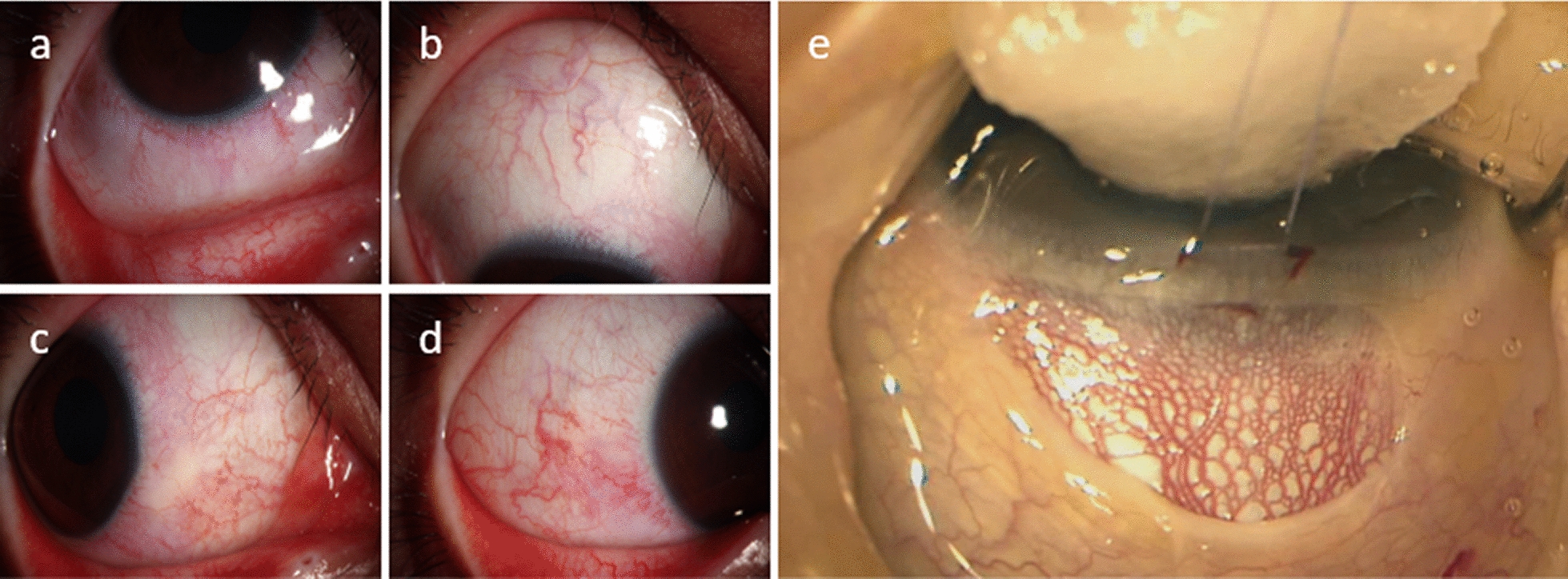
Conjunctival vascular malformation observed under slit lamps (**a**-**d**), episcleral vascular malformations observed intraoperatively (**e**)

**Fig. 2:**
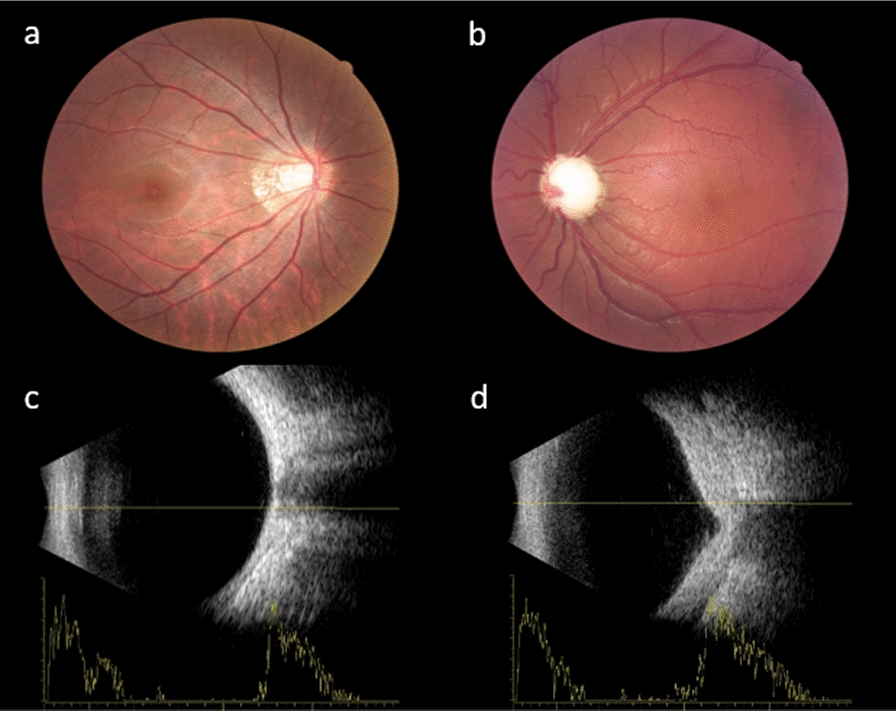
Fundus photography (**a**, **b**) and ocular B-scan ultrasound (**c**, **d**) of a patient with Sturge-Weber syndrome complicated with choroidal capillary malformation. Pronounced tomato-red fundus (**b**) and diffuse choroidal thickening (**d**) in the left eye can be observed

SG of SWS may cause numerous ocular manifestations (summarized below):

1. Anterior segment vascular malformations: conjunctival vascular malformations can be directly observed under a slit-lamp examination while in certain areas the deeper vessels noted sub-conjunctivally are episcleral vascular malformations. However, the reticular pattern of the diffuse episcleral vascular malformations is best visualized intraoperatively after Tenons dissection (Fig. 1). Elevated EVP in patients with SG may manifest as marked dilatation of the episcleral vessels with increased density and reticular distribution. Dilated and tortuous conjunctival and scleral vessels even with spiral curvature, are pathognomonic for increased episcleral venous pressure gradients. The results of one study suggest that the severity of the episcleral vascular malformations and larger corneal diameters correlate with failure of trabeculotomy [27].
2. Glaucoma-related manifestations: SG also presents with glaucoma-related manifestations in the form of increased IOP, optic nerve damage, and visual field changes. In early childhood glaucoma, examinations can be performed under general anesthesia. The examinations include IOP (measured by applanation tonometer or contact tonometer), horizontal corneal diameter, corneal edema, central corneal thickness, cup-to-disc ratio and dilated fundus examination. In addition, ocular B-scan ultrasound can be used to roughly compare the axial length of the eyes to assist in the diagnosis of SG and monitor the progression of the disease [37]. In adults who are able to cooperate, a range of diagnostic techniques may be employed, including IOP measurement, ocular B-scan ultrasound, fundus photography, optic disc optical coherence tomography (OCT), and visual field assessment. Of note, when analyzing visual field defects in patients with SG, it is important to consider not only the possibility of SG, but also that SWS-related occipital lobe involvement may also lead to visual field defects [38].
3. Choroidal CM: dilated fundus examination and ocular B-scan ultrasound and can detect the presence of choroidal CM (Fig. 2). It has also been reported that SWS patients with combined choroidal CM are more likely to develop glaucoma [39]. The most common presentation is “tomato red” fundus, which was previously often referred to as “choroidal hemangioma”, and is now considered more appropriately described as “choroidal capillary malformation” [15, 40]. A significant reduction in choroidal thickness was observed in the post-mortem state, concurrent with the drainage of blood [41], which supports the hypothesis that the typical “tomato red” fundus observed in patients with SWS is caused by diffuse thickening of the choroid, rather than by cell proliferation. Enhanced depth imaging optical coherence tomography can quantitatively measure choroidal thickness. One study found that choroidal thickness increased in PWB affected eyes compared to normal eyes, and this trend was more pronounced in SG eyes [42]. The majority of contemporary researchers concur that observation therapy may be a viable option for asymptomatic cases of choroidal CM without subretinal fluid [43].
4. Choroidal and retinal effusions: the characteristic choroidal vascular malformation in SWS leads to abnormal local blood flow and increased vascular permeability, thereby facilitating the abnormal accumulation of fluid. The most common situation is when the patient receives filtering surgery. The rapid reduction of IOP results in an increase in the venous pressure difference between the choroid and the anomalous venous system of SWS. This results in increased exudation and an increase in choroidal effusion, retinal effusion, or even choroidal detachment or retinal detachment [44]. Surgery is contraindicated for exudative retinal detachment. Some earlier studies suggested that photodynamic therapy and intravitreal injection of anti-VEGF drugs may be treatment options in cases of SWS with choroidal CM [43, 45]. Recently, mainstream opinion held that observation is the main treatment [46]. Most exudative retinal detachments can recover spontaneously, which necessitates a combination of patience from both medical professionals and patients. Moreover, the administration of oral propranolol during the perioperative period is thought to minimize the development of choroidal effusion after glaucoma surgery in SWS [47].
5. Strabismus and amblyopia: may be attributed to the advanced visual impairment caused by SG during growth and development [48, 49].

#### Cutaneous presentations

**Fig. 3:**
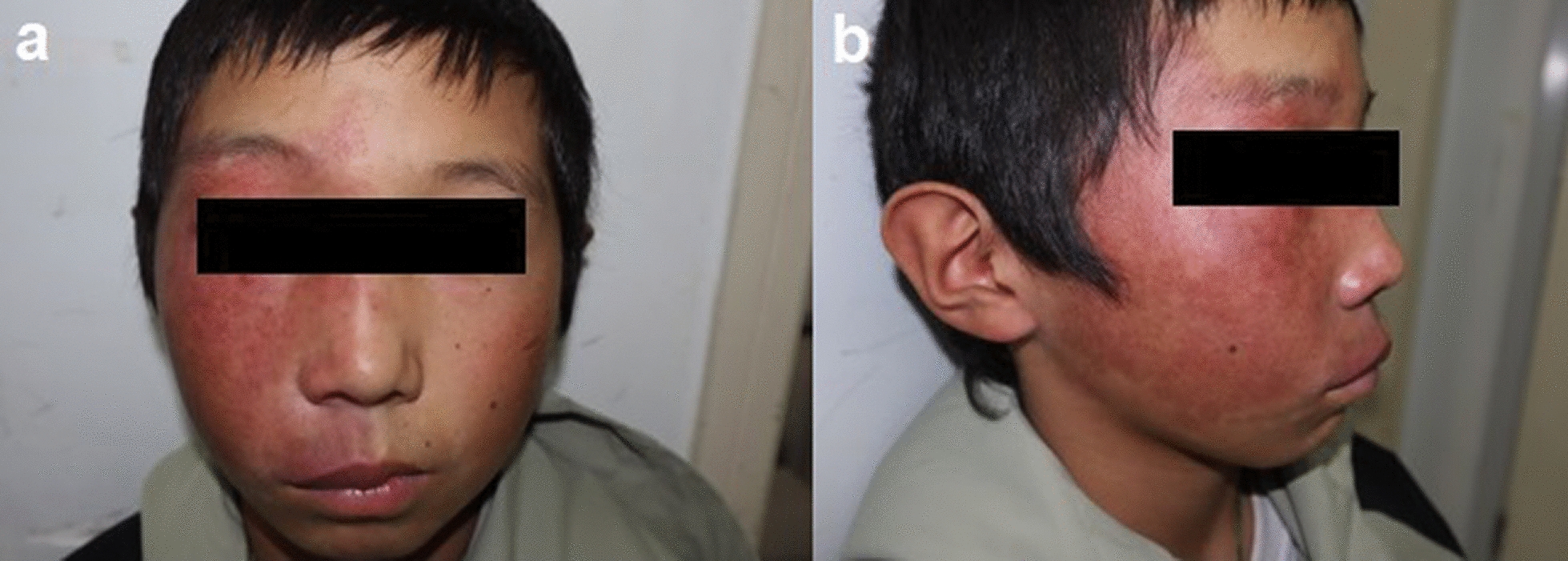
Frontal (**a**) and lateral (**b**) photographs of a patient with Sturge-Weber syndrome, showing right-sided port-wine birthmark

PWB is the result of differentiation-impaired endothelial cells with a progressive dilatation of blood vessels [50]. Initially, it manifests as a pink or red patch, which then appears to darken or develop soft tissue hypertrophy or proliferative nodules over time [51]. In most cases, the cutaneous manifestation of SWS presents as facial PWB, there are also scattered cases with presence of PWB in the trunk or extremities, or in the absence of any visible PWB [32, 52]. Previous studies have indicated that facial PWB matches the trigeminal nerve (cranial nerve V) distribution. SWS is associated with the involvement of the ophthalmic branch of the trigeminal nerve (V1). However, the extent of PWB in SWS often does not reach or exceed the median line and is sometimes found in the scalp which is not innervated by the trigeminal nerve. Other studies have suggested that the distribution of PWB in SWS is more consistent with the pattern of distribution of facial embryonic blood vessels, prefrontal PWB in an imaginary line between the lateral canthus of the eye and the top of the ear (including the upper eyelid) is highly correlated with the development of SG (Fig. 3) [3, 53].

Furthermore, SG has been associated with PWB distribution patterns. In cases of SG, the eye involved is usually ipsilateral to the PWB. Additionally, some findings suggest that right-sided and bilateral PWB may be more likely to be combined with SG [54]. The risk of developing SG is higher when PWB involves both the upper and lower eyelids than when it affects only the upper eyelid. In a study of 100 children with PWB, the incidence of glaucoma was 72% in those with involvement of both eyelids, compared to 21% in those with only the upper eyelid involvement [2]. These findings confirm that children with PWB in any part of the forehead should have early ophthalmologic examination [55].

#### Neurological presentations

Neurological abnormalities in SWS include seizures, migraine headaches, fluctuating hemiparesis, with the most common being seizures [56]. Although seizures can continue to occur throughout life, it often starts in the first 2 years of life, occurring earlier in those with bilateral or extensive unilateral hemispheric disease [57]. SWS brain involvement is marked by a leptomeningeal vascular malformation on contrast enhanced magnetic resonance imaging (MRI) images. Some studies agree that a normal neurological exam, no history of seizure onset, and negative MRI image with contrast after 1 year of age can generally exclude SWS brain involvement [58].

## Treatment

### Drug

The drugs used for SG are summarized in Table 2.

**Table 2:** Summary of clinical studies of drugs for the treatment of Sturge-Weber syndrome secondary glaucoma

Drug	Author	Number of eyes involved	Follow-up (months)	Result
Propranolol	Wygnanski-Jaffe et al. [60]	4	3	One patient successfully controlled (a temporary hypotensive effect after one week)
Latanoprost	Ong et al. [67] Altuna et al. [72]	14 18	12 6	50% successfully controlled 16.7% (3/18) successfully controlled
Netarsudil	Kaufman et al. [75]	6	25	Average IOP reduced by about 30%

*IOP* = intraocular pressure

#### Beta blockers

The use of topical β-blocker eye drops has been demonstrated to be an effective method of controlling elevated IOP in patients with glaucoma. In addition, the oral β-blocker propranolol is the drug of choice for the treatment of cutaneous hemangiomas in children [59]. A prospective study demonstrated that systemic use of propranolol had a short-acting IOP-lowering effect, likely due to vasoconstriction. In this study, 2 mg/kg oral propranolol was administered to children with SG and a decrease in IOP was observed in all four children after one week, but the effect gradually diminished thereafter. At 1 month, IOP increased again in three patients, but the cornea remained clear [60]. This effect can be utilized to control the IOP perioperatively. Furthermore, the administration of propranolol in the perioperative period has been demonstrated to reduce the extent of choroidal effusion and relieve exudative retinal detachment [47, 61]. However, unlike how propranolol acts on infantile hemangioma, the effect of oral propranolol on choroidal CM or PWB of SWS is not significant. In Krema et al.’s case report, two patients of SWS with PWB and choroidal CM showed no change in PWB color or choroidal thickness after 6 months of oral propranolol [62]. Previous studies have shown that the GNAQ R183Q mutation was present in the PWB and choroidal vascular bed of SWS patients while the immunofluorescence staining for the infantile hemangioma marker GLUT1 was negative [63, 64]. It can be hypothesized that one of the reasons for this may be that propranolol acts on the mitogen-activated kinase (MAPK) pathway, which is hyperactive in infantile hemangiomas, but shows moderately elevated signaling activity in the MAPK pathway in cells with GNAQ mutations [4, 62, 64].

#### Prostaglandin analogs (PGA)

Opical PGAs can increase matrix metalloproteinases (MMP) activity and extracellular matrix (ECM) turnover in aqueous outflow tissues, leading to tissue remodeling that enhances uveoscleral outflow [65]. Latanoprost, a nitric oxide-donor prostaglandin F2a analog, may reduce IOP by bypassing the abnormal trabecular tissue in patients with SG. In theory, late-onset SG may be less effective due to elevated EVP. However, it may also be related to the location of vein distribution. The results of previous studies have shown that latanoprost significantly reduces IOP in eyes with late-onset SG, but not in those affected with early-onset eyes [66, 67]. In Uva’s study, only 30% of patients with PCG have an IOP-lowering effect with latanoprost, the factors related to the failure include young age at PCG presentation [68]. It has been proposed that repeated stretching and pulling on the trabecular meshwork reduces the function of MMP and alters ECM [69, 70]. Both PCG and early-onset SG can manifest as buphthalmos, which may reduce the level of effective MMP applied by latanoprost, leading to a further increase in IOP. Besides, latanoprost has been confirmed to increase trabecular meshwork outflow, and abnormal anterior chamber angle development in early-onset SG may also contribute to the poor efficacy of latanoprost [71]. Moreover, latanoprost may cause the filling of the episcleral vessels, which increases the chance of surgical failure [72]. The interaction of increased uveoscleral outflow from latanoprost with elevated EVP may also lead to choroidal effusion. Therefore, latanoprost should be used with caution in patients with SWS diagnosed with choroidal CM [73, 74].

#### ROCK inhibitors

Netarsudil is a rho-associated protein kinase (ROCK) inhibitor that lowers IOP by a variety of mechanisms, including lowering trabecular outflow resistance, reducing aqueous humor production (via inhibition of ATPase in Na^+^/K^+^-ciliary epithelial cells), antifibrotic effects and lowering the EVP. It is particularly unique in its ability to reduce EVP. In a small cohort study, Kaufman used 0.02% topical netarsudil for the treatment of SG, demonstrating that netarsudil was an effective therapeutic agent in SG with elevated EVP [75]. Nevertheless, there is a paucity of data from large-scale trials investigating the efficacy of ROCK inhibitors in lowering IOP in patients with SWS.

#### Acetazolamide

In certain cases, oral acetazolamide has been shown to control seizures in patients with SWS. This may be related to the fact that acetazolamide inhibits carbonic anhydrase isoenzyme activity, and thus prevent pH imbalance and altered gamma-aminobutyric acid (GABA) activity caused by carbonic anhydrase. The administration of oral acetazolamide in the perioperative period can be an effective means of achieving dual control of SWS patients with combined ocular hypertension and epilepsy.

### Surgery

SG is most commonly associated with ocular anatomical abnormalities. It is important to note that drug therapy alone is not always effective. SG patients often require additional surgical interventions, which are associated with higher risks and more postoperative complications. Therefore, surgical methods must be selected with great care.

Early-onset SG is primarily associated with anterior chamber angle deformity, hence trabeculotomy or goniotomy are considered the optimal initial surgical approach in this patient population [76, 77]. In patients with late-onset glaucoma, filtering surgery or implantation of a glaucoma drainage device is often the chosen approach [15]. The surgeries applied to SG are summarized in Table 3.

**Table 3:** Summary of different surgical procedures used for Sturge-Weber syndrome secondary glaucoma

Surgery	Author(s)	Number of eyes	Follow-up (months)	Results	Common complications
GT	Yeung et al. [78]	46	12	Good IOP control in 2%	Hyphema, anterior chamber angle synechiae, etc.
Trabeculotomy MAT	Wu et al. [81] Hu et al. [84]	34 13	36 54	Complete (conditional) success rate 66.0% (86.6%) Complete (conditional) success rate 76.2% (87.5%)	Hyphema, anterior chamber angle synechiae, etc.
Trabeculectomy	Sarker et al. [93]	20	22.95 ± 2.87	Complete (conditional) success rate 70%	Hypotony, subconjunctival fibrosis, filtering bleb failure, choroidal effusion, etc.
CTT VTT	Senthil et al. [24] Elwehidy et al. [89]	49 21	60 24	Complete (conditional) success rate 64% (89%) IOP decreased by 57.6%	Hyphema, choroidal effusion, etc. IOP spike, hyphema, etc.
AGV BVT Molteno tube Ex-Press	Kaushik et al. [92] Budenz et al. [94] Amini et al. [95] Wu et al. [96]	24 10 9 21	25.44 ± 10.44 35 6 36	Cumulative success rate 75% Good IOP control in all eyes Complete success rate 0% Complete (conditional) success rate 40% (70%)	Hypotony, tube malposition, tube obstruction, tube exposure, corneal decompensation, etc.
NPDS	Almobarak et al. [98]	12	83.0 ± 74.2	Cumulative success rate 66.6%	TDW perforation, iris prolapse, choroidal effusion and choroidal detachment, etc.
CTNS	Huang et al. [99]	22	22.3 ± 4.0	Complete (conditional) success rate 52.4% (85.7%)	Hyphema, TDW perforation, iris prolapse, choroidal detachment, etc.
Cyclodestructive procedure	van Emelen et al. [101]	7	48 to 60	Cumulative success rate 85.7%	Hypotony, conjunctival edema, uveitis, etc.

*IOP* = intraocular pressure; *GT* = goniotomy; *MAT* = ab externo microcatheter-assisted trabeculotomy; *CTT* = combined trabeculotomy with trabeculectomy; *VTT* = viscotrabeculotomy-trabeculectomy; *AGV* = Ahmed glaucoma valve; *BVT* = Baerveldt tube; *NPDS* = non-penetrating deep sclerectomy; *CTNS* = trabeculotomy-non-penetrating deep sclerectomy; *TDW* = trabeculo-Descemet window

#### Goniotomy

Goniotomy (GT) offers the advantage of being a relatively low-trauma procedure that can be performed multiple times. In Yeung’s study, 46 eyes of 42 patients with SG received GT, but the success rate was only 2% (1 out of 46 eyes) [78]. In Olsen’s study, 66% of eyes that initially underwent GT required repeated surgery [79]. The high failure rate may be attributed to the possibility that early-onset SG is associated with additional pathogenic mechanisms such as vascular factors. Consequently, although GT is not an efficacious initial pressure-lowering modality, it still can be employed to defer IOP elevation in patients with SG while other forms of glaucoma surgery should be considered at a later stage. GT surgery requires adequate corneal clarity, and the results suggest that lower baseline IOP and shorter axial length are significant predictors of surgical success [80]. Therefore, a comprehensive evaluation of the patient's corneal condition is essential prior to surgical intervention. The most common complications of GT are varying grades of hyphema in the postoperative period which can be resolved without intervention [80]. Moreover, miotic drugs (e.g., pilocarpine) should be employed both intraoperatively and postoperatively, to expose the trabecular meshwork intraoperatively and to minimize the risk of anterior chamber angle synechiae postoperatively [15, 80].

#### Trabeculotomy

Olsen et al.’s study showed that approximately half of eyes that initially underwent trabeculotomy required a secondary procedure [79]. Preoperative IOP, corneal status, pattern of distribution of the episcleral vascular malformations, and corneal diameter are prognostic indicators that influence the prognosis of SG patients undergoing trabeculotomy. When patients present with large corneas, high preoperative IOP, corneal edema, or extensive distribution of episcleral vessels in a reticular pattern with marked dilatation, these may indicate poor prognosis [27, 81].

Ab externo microcatheter-assisted trabeculotomy (MAT) has the benefits of accurate localization and the ability to incise a larger area of the trabecular meshwork compared to conventional trabeculotomy using a rigid probe. This procedure demonstrates better efficacy in pediatric glaucoma secondary to SWS [82, 83]. In Hu et al.’s study, the mean IOP decreased from 26.5 ± 5.3 mmHg to 16.5 ± 5.0 mmHg after MAT in 13 SG eyes with fewer postoperative complications [84]. One of the major causes of uncontrollable IOP after MAT is anterior chamber angle synechiae, which usually occurs at the early postoperative stage. The administration of pilocarpine for 3 months after surgery can reduce the occurrence of this condition [84, 85]. As with GT, hyphema is easy to occur after MAT. Observation is usually used as an initial method and anterior chamber irrigation can be adopted if necessary [84].

#### Trabeculectomy

Trabeculectomy has been demonstrated to be an effective treatment modality for SG [86]. Complications may arise following a trabeculectomy, including shallow anterior chamber, hypotony, subconjunctival fibrosis, and filtering bleb failure, amongst others. Intraoperative hypotony can be avoided by tight flap closure and the use of an anterior chamber maintainer [86]. The intraoperative combination of antimetabolites such as mitomycin C (MMC) has been shown to reduce subconjunctival fibrosis, increasing the long-term success rate of the surgery. Barbosa reported a case in which MMC in combination with trabeculectomy resolved a patient with SWS who had a combined subretinal effusion. This may be related to increased aqueous humor filtration due to filtering surgery, which reduces VEGF levels, inflammatory cascade response, and consequent vascular leakage [87]. In addition to MMC, trabeculectomy can be combined with other implants to minimize subconjunctival fibrosis formation and filtration bleb complications. In Mohamed’s study, trabeculectomy combined with the biodegradable collagen implant Ologen in SG patients demonstrated a comparable IOP-lowering effect with MMC along with a reduced incidence of postoperative complications [88].

#### Combined trabeculotomy with trabeculectomy (CTT)

Trabeculotomy establishes a connection between the anterior chamber and Schlemm's canal, while trabeculectomy creates a link between the anterior chamber and the subconjunctival space. This combined procedure overcomes EVP and creates a new pathway for the aqueous humor to flow out. In Senthil et al.’s study, 49 patients with SG underwent the first CTT. The qualified success rate was 98% at 1 year and 89% at 5 years. However, a high surgical failure rate was observed in patients with large cup-to-disc ratios [24]. Elwehidy et al. introduced viscotrabeculotomy-trabeculectomy (VTT) in addition to CTT surgery, in which high-viscosity sodium hyaluronate was injected into Schlemm’s canal before the trabecular incision. The study showed that the IOP-lowering effect of the VTT group was more pronounced than that of the CTT group at 1 month and the incidence of complications such as shallow anterior chamber, choroidal detachment and hyphema was lower than that of the CTT group, especially in the case of hyphema. This is likely attributable to the spatial maintenance effect of viscoelastic agents and the increase in blood tamponade. However, it should be noted that IOP spikes are also more likely to occur after VTT than CTT due to the residue of viscoelastic agents in anterior chamber [89].

#### Implantation of a glaucoma drainage device (GDD)

Patients with SG are characterized by high EVP. Implantation of a GDD can bypass this pressure and create a new channel for external drainage of the aqueous humor. However, the GDD surgery has relatively more postoperative complications such as hypotony, tube malposition, tube obstruction, tube exposure and corneal decompensation [90]. The most common types of glaucoma drainage devices include the Ahmed glaucoma valve (AGV), the Baerveldt tube (BVT), the Molteno tube, and the Ex-Press drainage.

In Beck et al.’s study, 46 eyes of 32 patients underwent GDD (both AGV and BVT), while 24 eyes of 19 patients underwent trabeculectomy combined with MMC. The cumulative success rate in the GDD group was 87%, compared with 36% in the trabeculectomy group at 12 months. However, it requires more postoperative management, with the most common issue being drainage device displacement, which requires repositioning surgery in 65% of cases [91].

##### Ahmed glaucoma valve implantation

AGV is a unidirectional valvular drainage implant. In contrast to other drainage implants, AGV does not require internal occlusion or external ligation and can prevent postoperative hypotony. The cumulative success rate of 24 eyes that underwent AGV surgery was 75% [92]. Sarker et al. divided 40 patients with SG into two groups: the AGV group and the trabeculectomy with combined MMC group. Postoperative IOP decreased in both the AGV and trabeculectomy groups although the difference between the two groups was not statistically significant [93].

##### Baerveldt tube implantation

The advantage of BVT is that the use of an intraluminal stent and ligation of the tube intraoperatively can avoid an acute reduction in IOP [86]. To minimize the risk of intraoperative hemorrhage, Budenz et al. performed a two-stage Baerveldt glaucoma implantation in nine eyes. This procedure was found to be effective in reducing IOP during follow-up visits [94].

##### Molteno tube implantation

The Molteno tube is a drainage device that transfers aqueous humor from the anterior chamber to an equatorial collecting pool located posteriorly. It mechanically expands the space under the Tenon's capsule as more aqueous humor is drained and prevents fibrous obstruction by bypassing the anterior conjunctiva and inserting the posterior outlet of the tube directly into the collector. Nine eyes of seven patients with SG were included in Amini et al.’s prospective study, with a relative success rate of 78% at 1 year and 43% at the last follow-up timepoint. However, follow-up after 6 months postoperatively yielded no cases with complete success and no improvement in visual acuity [95]. Postoperative complications included choroidal effusion requiring surgical drainage, cataracts, and retinal detachment. The results of this study indicate that initial Molteno tube implantation has a limited success rate but a relatively high complication rate in pediatric eyes with SG due to SWS.

##### Ex-Press drainage implantation

The Ex-Press can be employed as a standalone procedure or in conjunction with MMC for trabeculectomy. In Wu et al.’s study, IOP in 21 SG eyes which underwent Ex-Press drainage implantation decreased by about 40% at the 3 months follow-up, but the majority of eyes required additional intervention during long-term follow-up [96]. However, it is worth mentioning that the Ex-Press shunt implantation showed a relatively high level of safety [97]. In the study, 11 eyes were diagnosed with diffuse choroidal CM, but only two eyes developed choroidal effusion and exudative retinal detachment and one eye developed shallow anterior chamber after surgery, all of which were successfully treated with conservative treatment [96].

#### Nonpenetrating glaucoma surgery

Given that SWS is associated with elevated pressure gradients within the vascular system of the choroid, a precipitous decline in IOP subsequent to filtering surgery increases the likelihood of choroidal detachment, suprachoroidal hemorrhage, and retinal detachment. To avoid these complications, non-penetrating procedures may be a better option for patients with SG.

Non-penetrating deep sclerectomy (NPDS) is a non-penetrating glaucoma procedure that allows for the gradual drainage of aqueous humor through the trabeculo-Descemet window (TDW). Aqueous humor from the anterior chamber can be filtered through the TDW into the sub scleral lake, thereby bypassing the high EVP of SWS. Furthermore, this procedure avoids sudden hypotony during and after the filtering surgery. In Almobarak et al.’s study, the success rate of deep sclerectomy was 67%. Intraoperative complications include TDW perforation, in which case the scleral flap should be carefully separated while maintaining the stability of the anterior chamber. When the TDW is perforated along with iris prolapse, peripheral iridectomy should be performed, and the surgery can convert to a trabeculectomy. Eyes with TDW perforation and iris prolapse are more likely to have choroidal effusion and choroidal detachment postoperatively [98].

Consequently, some studies have employed the trabeculotomy-non-penetrating deep sclerectomy (CTNS) on the aforementioned grounds. This procedure allows the aqueous humor to enter the Schlemm's canal directly from the anterior chamber through the trabeculotomy incision, or to drain into the suprachoroidal space and subconjunctiva through the TDW. It can be drained into the suprachoroidal space, scleral connective tissue, and subconjunctival space through the TDW, taking into account both the hypotensive effect and safety. In Huang et al.’s study, CTNS was performed on 22 eyes, with an overall success rate of 19 eyes at the last follow-up visit. Meanwhile, the trabeculo-Descemet’s membrane should be retained as thin as possible to ensure that the aqueous humor gradually seeps out. A 25 mg/mL of 5-fluorouracil sponge can be placed under the conjunctival sac and scleral flap for 3 min after the superficial scleral flap of CTNS was created for anti-scarring treatment [99].

#### Cyclodestructive procedures

Cyclodestructive procedures can be defined as an intermediate or an additional procedure in select cases, such as failed prior glaucoma surgery, advanced glaucoma with poor visual potential or family refusal of incisional surgery [100]. In van Emelen's studies, six of the seven SG patients who underwent the cyclodestructive procedure were able to control IOP at mean follow-up of 4–5 years [101]. According to Parsa, the presence of preoperative conjunctival edema in a patient with SWS indicates that episcleral venous leakage has exceeded the lymphatic drainage capacity. This condition may indicate that the pressure-reducing effect of filtering surgery is not significant and cyclodestructive procedures are a potential treatment option in such cases [15]. However, it is important to note that cyclodestructive procedures have a greater hypotensive effect and a higher risk of intraoperative and postoperative complications such as choroidal detachment. To prevent the hypotony, the choice of treatment parameters should be crucial [100].

#### Prophylactic sclerectomy

In order to avoid complications such as hemorrhage and postoperative choroidal detachment, it has been suggested that prophylactic sclerectomy be performed prior to pressure-reducing surgery or during filtering surgery in SG [102]. In Bayoumi et al.’s study, 11 eyes with SG were performed prophylactic sclerectomy at the time of CTT. The result showed the IOP reduction in around 90% and reversal of optic nerve cupping in 73% of the operated eyes [22]. Nevertheless, it has also been proposed that postoperative choroidal and subretinal effusions are transient phenomena that resolve spontaneously without surgical intervention. In a retrospective study, 17 patients underwent glaucoma filtering surgery without prophylactic sclerotomy, and no serious intraoperative complications occurred. However, postoperative choroidal detachment was noted in six eyes that resolved with conservative management and did not require surgical intervention [103]. Beyond that, in cases where a patient presents with extensive episcleral vascular malformations, the likelihood of complications such as bleeding is increased. Consequently, the necessity for prophylactic sclerectomy should be carefully assessed.

## Perioperative management

SWS is a syndrome with cerebral-facial and ocular involvement. Therefore, management of multiple disciplines and special care during ocular perioperative periods are crucial for reducing the complications and improving the success rate of surgery. Long-term multidisciplinary follow-up also helps to improve the overall prognosis of SWS patients.

### Preoperative care

#### Systemic

It is particularly important to be mindful of the risk of seizures during the perioperative period. Due to the high incidence of seizures in SWS and the potential deleterious effects of seizures in the developing brain during the developmental phase, the use of preventative anti-seizure treatment in SWS can be used and an individualized emergency plan should be developed in advance to include treatment with benzodiazepines [104]. If migraines occur, some suggest that sleep, hydration, ibuprofen use and anti-emetics are effective [105]. For headache and migraines prophylaxis, the use of anti-seizure medications, including valproic acid, gabapentin and topiramate have been suggested [104]. It should also be noted that studies have reported that the use of topiramate may increase the risk of glaucoma [106]. Although an earlier study on its use for seizure treatment in SWS did not show increased incidence of glaucoma, it is still recommended to use topiramate with caution in SWS [107].

#### Ocular

A comprehensive assessment of the patient's ocular condition is essential prior to surgical intervention including using the ocular B-scan ultrasound and dilated fundus examination to detect the presence of choroidal CM. It is recommended to use oral propranolol under the guidance of the cardiologist to reduce preoperative IOP and the incidence of postoperative complications such as choroidal effusion. Currently, if the general condition permits, most clinical applications is a dose of 2 mg/kg of body weight daily during the perioperative period (1 week preoperatively and continued for 6–8 weeks postoperatively) [24]. Additionally, antibiotic eye drops should be administered routinely to control infection.

### Intraoperative measures

Due to the high incidence of complications in patients with SWS, the most significant consideration in glaucoma surgery is slow decompression of the globe to prevent complications such as hypotony or choroidal detachment. Intraoperative application of high-viscosity sodium hyaluronate, tight flap closure and gentle movements by the surgeon are beneficial for maintaining the stability of the anterior chamber. In NPDS, a thinner TDW should be preserved as much as possible while carefully separating the scleral flap. Furthermore, the use of miotic agents intraoperatively can assist in the exposure of the trabecular meshwork structures and expand the surgical field of view.

### Postoperative monitoring

Topical steroids and non-steroidal anti-inflammatory drugs should be routinely used postoperatively to reduce the inflammatory response. Depending on the type of surgery, special medications may be required, such as miotic agents to prevent angle synechiae after GT or trabeculotomy [80]. Additionally, postoperative complications including hyphema, choroidal effusion, and IOP spike, should be monitored. Slight hyphema can be self-absorbed without special treatment, and anterior chamber irrigation can be performed if necessary [84]. Postoperative IOP spike may require withdrawal of topical steroids, repeated anterior chamber paracentesis or addition of IOP lowering drugs. Regular ocular B-scan ultrasound and OCT can detect the occurrence of complications such as choroidal effusion, choroidal detachment or retinal detachment. Observation therapy is the mainstay of treatment, other treatments like oral medications can be given in selected cases [47]. Besides, rigorous amblyopia therapy results in good outcomes in terms of vision at an early age [108].

### Multidisciplinary follow-up

SWS involves a variety of disciplines including neurology, dermatology and ophthalmology. Long-term cross-disciplinary follow-up is of great significance for improving the patients’ prognoses. As patients with SWS have a life-long risk of glaucoma, regular ocular examinations are essential for monitoring the occurrence and development of glaucoma [104].

Seizures are also a lifelong disease, and it is currently believed that epilepsy should be treated aggressively with a goal of obtaining and maintaining complete seizure suppression as far as possible [107, 109]. The majority of patients with SWS present with cosmetic dermatological needs. To date, there is no evidence that superficial skin laser treatment of facial PWB is associated with increased IOP or the elevation of pre-existing high IOP [110–112]. However, it is still recommended that patients with SWS in infancy and early childhood refrain from skin interventions until the collateral circulation pathways in the brain have improved and the critical period of brain maturation has passed, at least in the first year of life [15]. Patients with SWS often have a decline in cognitive function [113], suggesting that family members and clinicians should pay more attention to psychological concerns in patients with SWS, particularly in children undergoing the process of personality development.

Taken together, the establishment of a multidisciplinary referral and comprehensive management platform is beneficial for the early detection of other systemic diseases and the implementation of timely intervention in patients with SWS.

## Conclusion

The treatment of SG is challenging undertaking and requires the input of a physician with extensive experience. The distribution pattern of PWB can be used as an indicator when screening for glaucoma, especially in those with the condition involving the forehead. SG can be managed with medications or surgical intervention, however the incidence of surgical complications is higher due to the anatomical abnormalities of SWS. Thus, safer non-penetrating surgical procedures are available.

Somatic mutations in SWS give rise to a number of molecular processes that can be modulated by biologically active compounds to ameliorate the pathological consequences of SWS patients. Further mechanistic studies can be focused on some biological targets for SWS including carbonic anhydrase inhibitors, acetylcholinesterase, GABA receptors, and the hypoxia-inducible factor − 1α and 2α [114] or the pathogenic pathways of SWS, such as MEK pathway and YAP inhibitors[4, 11].

Due to the low prevalence of SWS, most studies have been unable to enroll a large number of cases. The establishment of integrated standardized database and mature multidisciplinary consultation referral model can assist clinicians in comprehensively recognizing and treating SWS patients.

## Data Availability

All data generated or analyzed during this study are included in this published article.

## Abbreviations

IOP: Intraocular pressure
SG: Sturge-Weber syndrome secondary glaucoma
SWS: Sturge-Weber syndrome
PWB: Port-wine birthmark
EVP: Episcleral venous pressure
VEGF: Vascular endothelial growth factor
CM: Capillary malformation
PGA: Prostaglandin analogs
MMP: Matrix metalloproteinases
ECM: Extracellular matrix
ROCK: Rho-associated protein kinase
GABA: Altered gamma-aminobutyric acid
YAP: Yes-associated protein
PCG: Primary congenital glaucoma
OCT: Optical coherence tomography
MRI: Magnetic resonance imaging
GT: Goniotomy
MAT: Ab externo microcatheter-assisted trabeculotomy
MMC: Mitomycin C
CTT: Combined trabeculotomy with trabeculectomy
VTT: Viscotrabeculotomy-trabeculectomy
GDD: Glaucoma drainage devices
AGV: Ahmed glaucoma valve
BVT: Baerveldt tube
NPDS: Non-penetrating deep sclerectomy
TDW: Trabeculo-descemet window
CTNS: Trabeculotomy-non-penetrating deep sclerectomy
